# Flexor Tendon Entrapment at the Malunited Base Fracture of the Proximal Phalanx of the Finger in Child: A Case Report

**DOI:** 10.1097/MD.0000000000001408

**Published:** 2015-09-04

**Authors:** Young-Keun Lee, Soojin Park, Malrey Lee

**Affiliations:** From the Department of Orthopedic Surgery, Chonbuk National University Hospital, Jeonju (Y-K L, M L); Graduate Scool of MOT/ Sogang Institute of Advanced Technology, Sogang University, Seoul, Korea.

## Abstract

The proximal phalangeal base is the most commonly fractured hand bone in children. Such fractures are rarely reported as irreducible due to flexor tendon entrapment. Here, we describe a patient who sustained a malunited fracture on the right fifth finger proximal phalanx with flexor tendon entrapment after treatment with closed reduction with K-wires fixation.

A 13-year-old patient came to the clinic following a bicycle accident 6 weeks ago. He presented with flexion limitation in his small finger on the right hand. During physical examination, the patient felt no pain, and the neurovascular structures were intact. However range of motion (ROM) in his small finger was not normal. Plain radiographs displayed a Salter–Harris type II fracture of the small finger proximal phalanx base and volar angulation with callus formation. During the operation, it was established that the flexor digitorum superficialis (FDS) around the fracture had a severe adhesion, whereas the flexor digitorum profundus (FDP) was entrapped between the volarly displaced metaphyses and the epiphyses and united with the bone. We removed the volarly displaced metaphyses and freed FDP and repaired the A2 pulley. The bone was anatomically fixed with K-wires. In the treatment after the operation, on the 2nd day, the patient was permitted the DIP joint motion by wearing a dynamic splint.

At the 12-months follow-up, the patient had regained full ROM with no discomfort and the proximal phalanx growth plate of the small finger closed naturally with normal alignment.

When treating a proximal phalangeal base fracture in children, the possibility of flexor tendon entrapment should be considered and should be carefully dealt with in its treatment.

## INTRODUCTION

The proximal phalanx is a common pediatric hand fractures.^1^ Most of these fractures may be treated using closed methods, which require immobilization for 3 to 4 weeks. However, open reduction and internal fixation may be observed in widely displaced fractures with interposition of soft tissue or unstable fractures.^2^ Such fractures are rarely reported as irreducible, due of flexor tendon entrapment.^3–5^ Harryman II et al^4^ reported on a 12-year-old female with physeal phalangeal fracture with flexor tendon entrapment. In addition, they asserted that treatment required early recognition to avoid further injury, open identification of the tissue derangement, and careful reduction to restore normal function. Woo et al^6^ reported on their case that call attention to the possibility of entrapment of flexor tendons in proximal phalangeal shaft fractures, especially when the mechanism of injury is crushing and the radiographic examination shows volar angulation deformity of the fracture fragments.

Here, we describe a patient who sustained a malunited fracture on the right hand of the small finger proximal phalanx with flexor tendon entrapment after treatment with closed reduction with K-wires fixation.

## CONSENT

The patient signed informed consent for publication of this case report and any accompanying image. The ethical approval of this study was waived by the ethics committee of Chonbuk National University Hospital, because this study was case report and the number of patients was <3.

## CASE REPORT

A 13-year-old patient came to the clinic following a bicycle accident 6 weeks ago. He presented with flexion limitation in his small finger on the right hand. Previously, he had received operation from another hospital with closed reduction and K-wires fixation because of a small finger fracture on the right hand. Four weeks after the surgery, the K-wires were removed.

**FIGURE 1:**
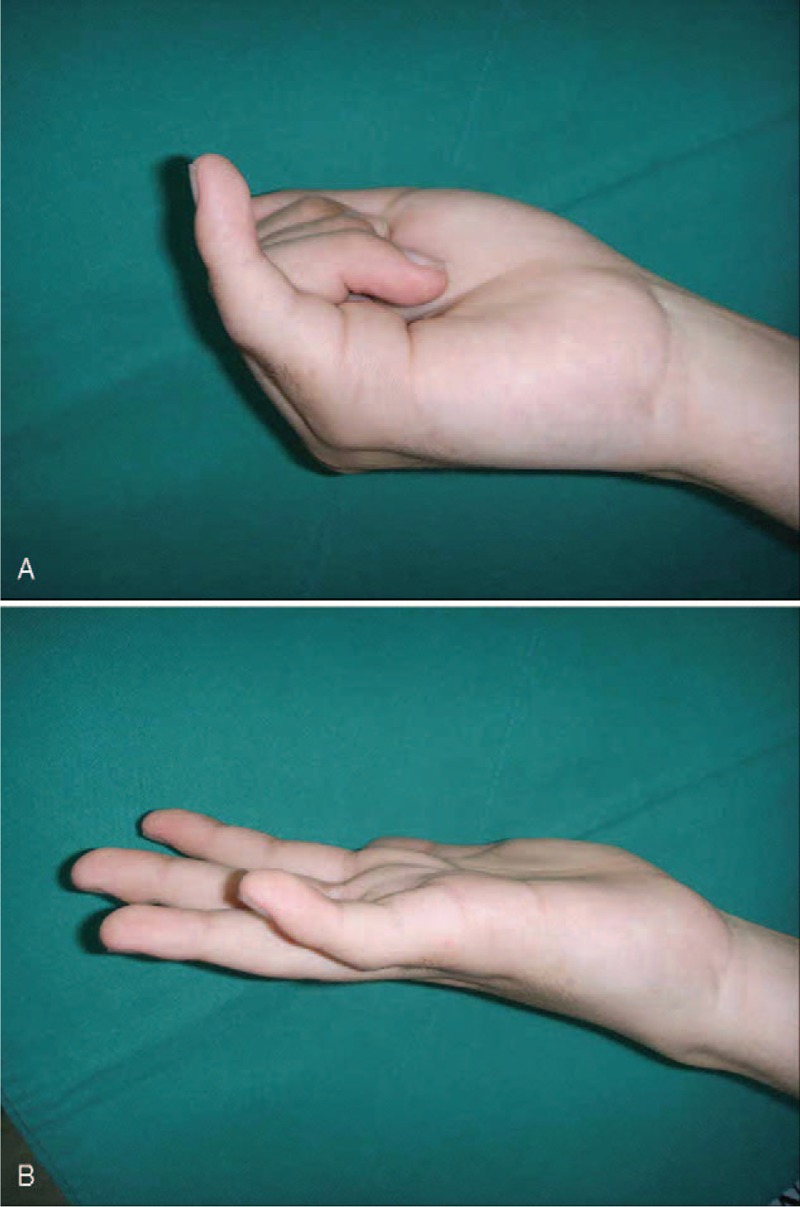
A–B. (A) Initial photograph after actively flex fingers shows some degree of flexion at MP joint. (B) Initial photograph after actively extend fingers shows extension of MP joint, but there is no range of motion of PIP and DIP joints compared to figure (A).

**FIGURE 2:**
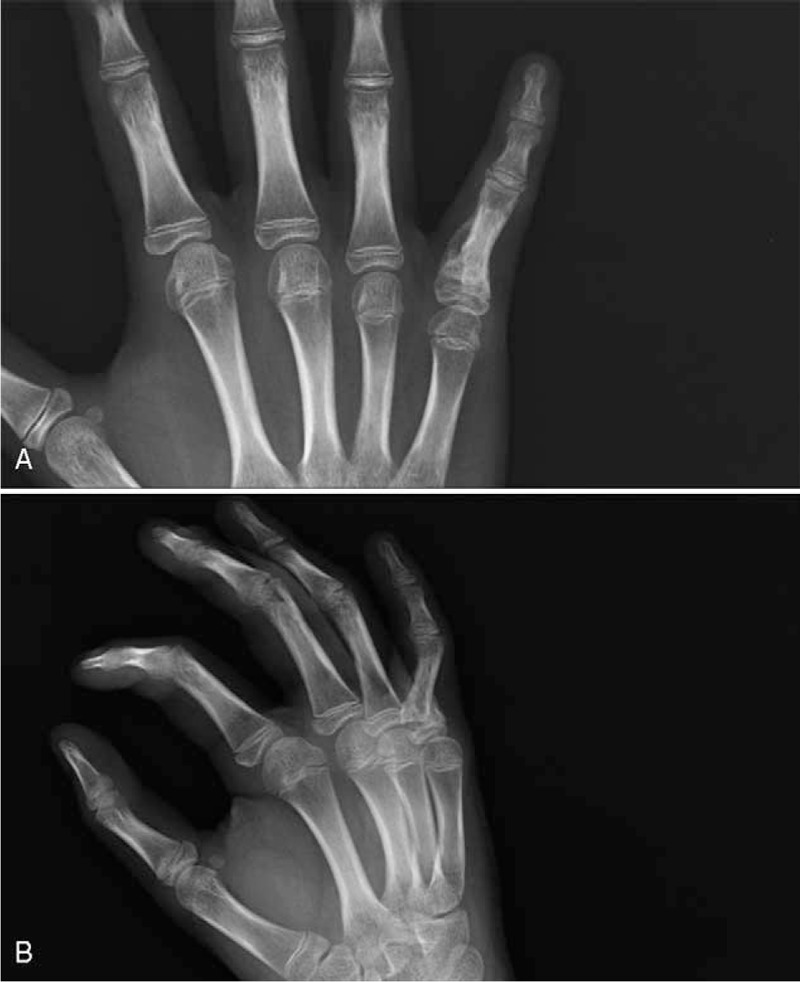
A–B. Preoperative AP (A) and oblique (B) radiographs demonstrate a Salter–Harris type II fracture of the small finger proximal phalanx base and volar angulation with callus formation.

During physical examination, the patient felt no pain, and the neurovascular structures were intact. However the range of motion (ROM) in his small finger was not normal, the metacarophalangeal (MP) joint had a 40 degree flexion, and 0 degree extension, the proximal interphalangeal (PIP) joint was fixed at 30 degree flexion and the distal interphalangeal (DIP) joint was fixed at 0 degree (Figure 1A and B). Plain radiographs displayed a Salter–Harris type II fracture of the small finger proximal phalanx base and volar angulation with callus formation (Figure 2A and B). This problem was due to adhesion of the flexor tendons at the malunion site of proximal phalanx base prompting the need for an operation.

**FIGURE 3:**
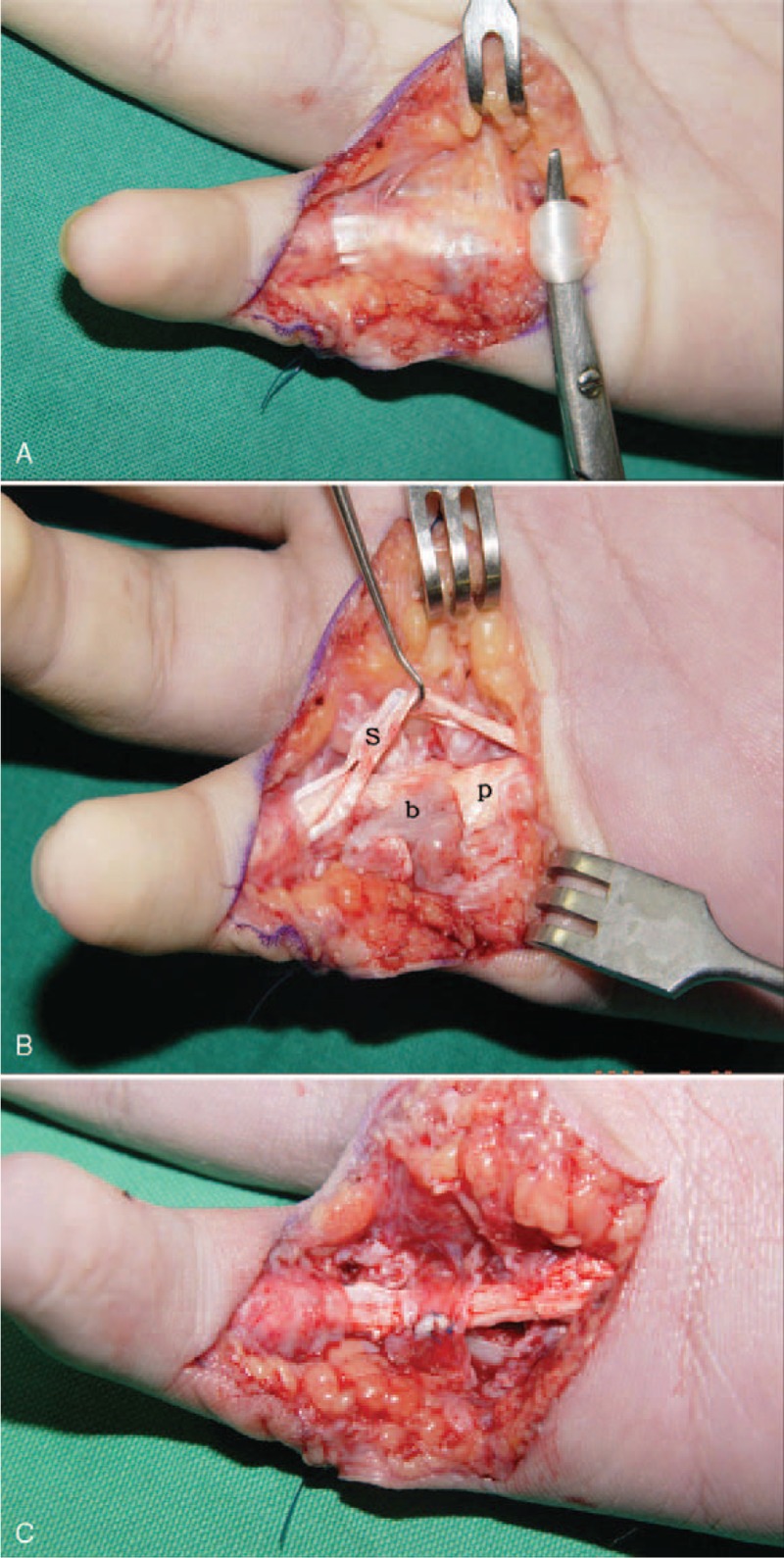
A–C. Intraoperative photographs show: (A) Flexor tendons adhesion at the fracture site. (B) FDP is interposed between the epiphysis and volarly displaced metaphysis and fracture is united. (b: bone, p: FDP, s: FDS) (C) A2 pulley is repaired after volarly displaced bone had been removed and FDP has been freed.

**FIGURE 4:**
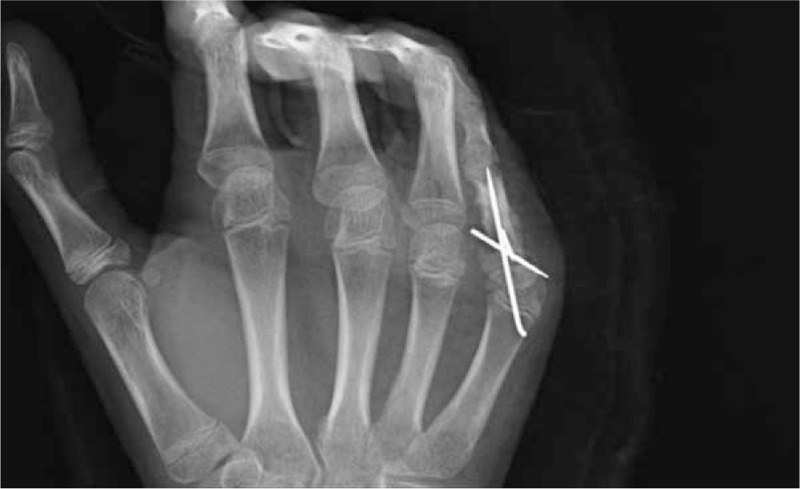
Postoperative oblique radiograph shows the correct alignment of the digit and 3 K-wires stabilize the fracture.

With the patient under general anesthesia, the fracture site was explored through a volar approach. During the operation, it was established that the flexor digitorum superficialis (FDS) around the fracture had a severe adhesion (Figure 3A), whereas the flexor digitorum profundus (FDP) was entrapped between the volarly displaced metaphyses and the epiphyses and united with the bone (Figure 3B). We removed the volarly displaced metaphyses and freed FDP and repaired the A2 pulley (Figure 3C). The bone was anatomically fixed with K-wires (Figure 4). In the treatment after the operation, on the 2nd day, the patient was permitted the DIP joint motion by wearing a dynamic splint. After 4 weeks, the K-wire was removed from the MP joint to allow motion about this joint, whereas the remaining K-wire was removed after 7 weeks.

**FIGURE 5:**
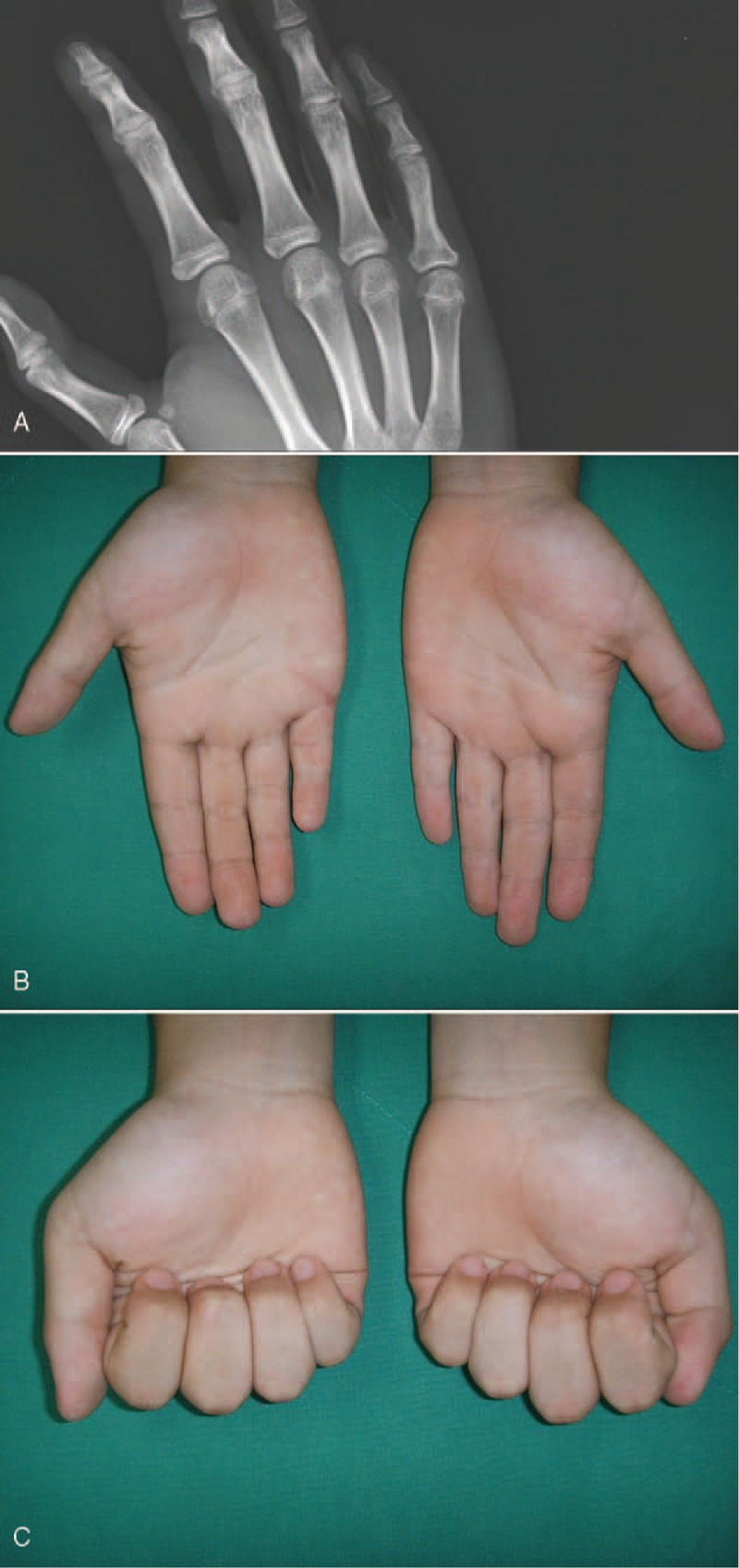
AC (A) An oblique radiograph obtained 12 months after operation shows the growth plate of proximal phalanx of small finger closed naturally. (B, C) Photographs obtained 12 months after operation show a normal range of motion and alignment of a right small finger.

At the 12-months follow-up, the patient had regained full ROM with no discomfort and the proximal phalanx growth plate of the small finger closed naturally with normal alignment (Figure 5A–C).

## DISCUSSION

Proximal phalanx fractures in children are usually extra-articular Salter–Harris type II injuries on the little finger.^7^ Previously reported causes of irreducible fractures that are secondary to soft tissue entrapment include fibrous tissue,^8^ extensor hood and periosteum,^8^ and flexor tendons.^3–5^ These fractures have some features in common.^5^ To start with, the injury mechanism is as a result of severe hyperextension that leads to dorsal angulation of the digit, moreover, the second, attempt of the closed reduction was unsuccessful, and third, the feeling of a rubbery resistance during manipulation indicates flexor tendon entrapment. Woo et al^6^ stress that it is necessary to check tendon gliding or motion of the interphalangeal joints using the wrist tenodesis effect under anesthetic block after operation. In the present study, the aforementioned features were never known because the first treatment was never involved and neither was the active range of motion exercise of finger after initial operation recommended.

In most cases, if the closed reduction method is unsuccessful for the treatment of phalangeal fracture, operation and K-wire fixation is usually the most preferred method. In the aspect of bone fixation, the anterior surface of the proximal phalanx forms the floor of the flexor tendon sheath and allows the tendon to glide properly with early return to normal function. Stable anatomical reduction and fixation is required.^4^

We consider that early ROM exercises of the finger with rigid bone fixation are necessary, freeing the flexor tendons at entrapped site, to avoid adhesion. Operation with 3 K-wires to fix the bone was used, and the DIP joint motion with a dynamic splint was permitted 2 days later.

## CONCLUSION

When treating a proximal phalangeal base fracture in children, the possibility of flexor tendon entrapment should be considered and should be carefully dealt with in its treatment.
