# Flexor Tendon Entrapment at the Malunited Base Fracture of the Proximal Phalanx of the Finger in Child: A Case Report: Erratum

**DOI:** 10.1097/01.md.0000472177.93758.5a

**Published:** 2015-09-25

In the article “Flexor Tendon Entrapment at the Malunited Base Fracture of the Proximal Phalanx of the Finger in Child: A Case Report”^1^, which appeared in Volume 94, Issue 35 of *Medicine*, Dr. Soojin Park's affiliation was not originally recorded. Dr. Park is affiliated with the Graduate Scool of MOT/Sogang Institute of Advanced Technology, Sogang University, Seoul, Korea. The article has since been corrected online.
